# Sex, Gender and Health: Mapping the Landscape of Research and Policy

**DOI:** 10.3390/ijerph19052563

**Published:** 2022-02-23

**Authors:** Lorraine Greaves, Stacey A. Ritz

**Affiliations:** 1 Centre of Excellence for Women’s Health, Vancouver, BC V6H 3N1, Canada; 2 School of Population and Public Health, Faculty of Medicine, University of British Columbia, Vancouver, BC V6T 1Z3, Canada; 3 Department of Pathology & Molecular Medicine, Faculty of Health Sciences, McMaster University, Hamilton, ON L8S 4K1, Canada; ritzsa@mcmaster.ca

**Keywords:** sex, gender, health, sex differences, sex and gender science, equity, gender transformative, equity, diversity & inclusion (EDI), intersectionality

## Abstract

Including sex and gender considerations in health research is considered essential by many funders and is very useful for policy makers, program developers, clinicians, consumers and other end users. While longstanding confusions and conflations of terminology in the sex and gender field are well documented, newer conceptual confusions and conflations continue to emerge. Contemporary social demands for improved health and equity, as well as increased interest in precision healthcare and medicine, have made obvious the need for sex and gender science, sex and gender-based analyses (SGBA+), considerations of intersectionality, and equity, diversity and inclusion initiatives (EDI) to broaden representation among participants and diversify research agendas. But without a shared and precise understanding of these conceptual areas, fields of study, and approaches and their inter-relationships, more conflation and confusion can occur. This article sets out these areas and argues for more precise operationalization of sex- and gender-related factors in health research and policy initiatives in order to advance these varied agendas in mutually supportive ways.

## 1. Introduction

The movement to build ‘sex and gender science’ inherently marries considerations of the body and society and illuminates the many sexed and gendered interconnected complexities attached to health and technology [1,2]. A transdisciplinary field, it is gathering momentum and becoming more recognized among a range of funders and health researchers. Its trajectory includes the development of increasingly complex concepts, theory, language and methods, all of which are crucial in meeting demands for tailored knowledge products made by consumer groups and community health advocates. Importantly, sex and gender science is critical for clinicians and health care providers who are pressed to provide more personalized care for patients, and policy makers who are interested in developing more equitable and representative population level health policy, regulatory frameworks and funding programs. 

Building sex and gender science in health is a multi-faceted enterprise, requiring several different but overlapping and complementary approaches. This article offers a conceptual outline of the scope of sex and gender science and its subsets, identifies related theoretical and policy enterprises, and articulates their interactions and intersections. These distinctions are increasingly required as the field expands and changes, both for experienced sex and gender science researchers, clinicians and policy makers, as well as those new to the fields, in order to build common understandings. We argue that in order to achieve equitable precision medicine and health care, we need to consciously build better sex and gender science, and that mapping its sub fields and relevant theoretical and policy approaches is required in order to achieve unity of purpose, clearer communications, effective implementation and better knowledge exchange.

Specifically, we argue that multiple approaches to building sex and gender science (such as researching sex and/or gender differences, sex- and gender-related factors, sex–gender interactions, or sexual and gender minority populations) are all beneficial and necessary; that sex and gender need to be integrated into intersectional approaches and vice versa in central, reciprocal and dynamic ways that enhance evidence building; and, that policy initiatives such as sex- and gender-based analysis plus (SGBA+) and equity, diversity and inclusion (EDI) are essential, but are in no way replacements for each other, or for sex and gender science, nor sufficient to ensure its overall development. Finally, we argue that while all of these elements are important and necessary and rely on each other, they do not replace each other, cannot be conflated, and ought not to be advanced singularly. Together, as a cumulative approach, they will improve the evidence base upon which health care, policy and programming rests, and ultimately contribute to gender-transformative approaches in health that improve health and gender equity simultaneously.

## 2. Background

All aspects of health research have long suffered from gender-blind approaches to research questions, design, methods, measures, analyses, reporting and implementation. Widespread gaps in knowledge have historically disadvantaged female bodies and women’s health, ultimately causing changes to research funding policy [3] and clinical trial development over the years [4,5,6,7,8,9] in attempts at redress, to respond to criticism, and to improve science. Other groups have also been consistently disadvantaged by hegemonic and prevailing research models and funding parameters, leading to uneven treatment of ethnic and racialized groups, pregnant and breastfeeding women, those with non-normative gender identities, and children and elderly people, resulting in significant gaps in knowledge that continue to reinforce disparities in health. While some of these gaps are slowly being addressed, the phenomenon of “we don’t know what we don’t know” regarding sex, gender and health persists. We must give ongoing attention to this ontological vacuum and continue to build newer and additional approaches and conceptualizations, in order to fully integrate sex and gender considerations in our understandings of health. 

Social movements and scientific advocacy have historically played key roles in developing concepts and approaches in generating evidence and integrating it into health care delivery and further health research. For example, highlighting sexist medical practices, omissions of females and women in clinical trials, adherence to the ‘male norm’ in medicine, and under researched women’s conditions and bodies can be attributed to the late 20th century women’s health movement, relying often on lived expertise to highlight gaps and injustices [10]. Similarly, civil rights movements and contemporary anti-racism groups such as Black Lives Matter have highlighted anti-racism [11], and sexual and gender minority groups and movements advocating for alternatives to heteronormative and gender normative health care have been important in promoting goals such as health and gender equity [12]. 

The push and pull of social movements and policy and regulation constantly evolves in the wider society. Within science, scientific advocacy has resulted in equity seeking perspectives, concepts, methods and reporting standards slowly being adopted by health research funders, policy makers, health care organizations, and government agencies. These mergers of activism and science are critical to understanding the history of sex and gender science as well as its future direction.

## 3. Research Approaches

The incorporation of sex and gender considerations into health research and policy is not a monolithic enterprise. Although there are certain shared goals and concepts, there are distinctive and evolving modes of engagement within the broader field [13]. We believe it is useful to continue to articulate these explicitly. Without such delineation, the field can sometimes feel fragmented, but by mapping this territory we can be more clear about how various perspectives and frameworks interact and complement one another to produce a fuller understanding of sex, gender and health.

We understand Sex and Gender Science as the overall enterprise that aims to generate, understand, and apply evidence related to sex and/or gender related factors and interactions in order to improve health. This specialty highlights the importance of understanding how sex- and gender-related factors (originating in both biology and sociology) affect human health and promulgate inter- and trans-disciplinary ideas about concepts, methods, measures and analyses of related evidence. For example, encouraging researchers to include sex as a biological variable (SABV) [14,15]) and gender as a sociocultural variable (GASV) [16] are examples of ongoing efforts to develop relevant measures. Robust sex and gender science is necessary to create evidence for supporting all areas linked to understanding the impacts of sex, gender and intersectional factors on health. In its absence, the ultimate goal of creating gender transformative approaches [17] that sensitively and effectively advance health *and* gender equity at the same time, will fail. 

Within the overall terrain of sex and gender science, there are several subgenres that describe different conceptual orientations to addressing sex and gender considerations:

- *Sex differences research* (sometimes also misleadingly labelled gender differences research) although more established, is a subset of sex and gender science. As its name implies, it is focused on identifying contrasting aspects of male and female bodies that impact health or bodily processes, conditions or diseases, responses to treatment or even longevity. *Gender differences research* is similar, in that it typically contrasts social and cultural experiences of men and women, boys and girls, and gender diverse people in order to derive knowledge. In both cases, the research design typically uses sex or gender categories themselves as the primary analytical framework, and as a result, these approaches are more likely to lead to the development of sex- or gender-specific actions, treatments, or interventions—that is, to suggest that men and women or males and females or gender diverse and fluid people require *different* treatments based principally on their sex/gender category.
- Research on *sex- and gender-related factors* draws our focus not to sex/gender categories per se, but rather to the components, factors and/or processes *associated with* sex or gender. This approach differs from sex or gender differences research in that it encourages identification of not just differences but also similarities, but more importantly, that it also focuses explicitly on the processes and elements of sex and gender that drive causal pathways. As a result, this approach is more likely to acknowledge the overlapping distributions and clusters of many sex- and gender-associated factors between males and females, and among men and women and gender diverse people, and is therefore more likely to lead to actions or interventions based on biological *mechanisms* or social *processes* rather than on a sex or gender category.
- Research focused on *sex/gender interactions* attends to the experiences of being a sexed body in a gendered social context, probing real world experiences of people and experiential impacts on bodies. These approaches recognize and identify ways in which gendered social experiences can influence biological phenomena (and vice versa), such as the ways that social experiences and behaviours like nurturance, competition, and assertiveness can change the expression of sex hormones like testosterone [18,19,20,21]. Conversely, such approaches can also examine how sexed characteristics (such as reproductive systems, or anatomical features) can shape work, socioeconomic opportunities and health [22].
- Broader *intersectional* approaches recognize that the operation of sex/gender-related factors is not homogeneous across populations or sex or gender categories. They consider the interactions between a range of characteristics (such as ability, age, sexual orientation, SES, race/ethnicity) in a sea of social and political processes (such as colonialism, sexism, racism, capitalism etc.) where gender as a social identity is usually included but not always deemed central [23]. Sex and gender science can benefit from intersectional approaches when *both* sex and gender and their interactions are studied in dynamic relation to these various characteristics and processes, are reciprocally integrated into multi-level intersectional analyses and models, and are not decentred, overlooked or replaced as considerations. In the context of sex and gender science, intersectional perspectives are important for elaborating the ways that the operation of sex and gender can be inflected by context.
- Research focussed on *sexual and gender minority populations* examines health and social issues of specific relevance for members of these communities. Given historic and ongoing oppression and marginalization of people based on non-normative sexual orientations, sexual identities, and behaviors as well as on non-normative gender identities and expression, there is much evidence documenting adverse health outcomes in both communities [24,25,26,27]. Sexual orientation and gender identity are not to be conflated in research or policy, as people with any gender identity can experience a range of sexual orientations, with myriad possibilities. Sex and gender science is developing data collection techniques and measures to advance the health of sexual minorities and gender minorities [26,28].

There are no hard lines between these approaches, nor are they mutually exclusive. However, by clarifying how these orientations differ and how they are related can help to situate different contributions to the overall sex and gender science enterprise and make their relationships, complementarity, and tensions more clear.

## 4. Policy Initiatives

Sex-, gender- and diversity-based analyses or gender-based analyses plus (S)GBA(+) as policy and program analysis tools have been favoured by some progressive governments and institutions over the past two decades, with varying levels of enforcement (see for example, Canada’s federal (S)GBA+ policy initiatives [29], auditor-general reports on their progress [30] and parliamentary committee reports [31]). SGBA+ is an analytic process that, when applied to health, involves assessing programs, policies, treatments, prevention, messaging and health promotion for differential impacts on people based on sex, gender and a range of diversity factors, with a view to tailoring such efforts to achieve more equity. GBA+ can be applied to any realm of human activity [29] while SGBA+ is specifically required in health and medicine [32]. SGBA+ is also useful in assessing research, as a tool that invites detailed review of research methods, data analysis and reporting and can be a component in assessing quality of studies, such as systematic reviews [33]. SGBA+ applies learning from both social and biological sciences, to generate speculation about potential effects and impacts. SGBA+ requires skill building, training, critical thinking skills, lateral thinking, speculation, and iterative considerations in assessing evidence gaps or potential differential impacts. Assessments of research design, data collection, analysis or reporting are increasingly applied by journals in shaping requirements for publishing research to facilitate reporting on sex and gender [34]). 

Equity, diversity and inclusion (EDI) initiatives have, more recently been promoted by governments, corporations, workplaces and educational institutions as initiatives to improve, change or reflect the population in the composition of a workforce or student body. EDI initiatives are policies and processes aimed at achieving equity in science, policy (and other pursuits) that focus on increasing inclusion of women, diverse, racialized, and minority groups as students, mentors and researchers. EDI includes goal setting, analytic processes, inclusion mechanisms and actionable goals. EDI initiatives often include concomitant processes for improving the climate for non-white, non- male, non-normative individuals and groups. Similar in impetus to late 20th century ‘affirmative actions’ intended to rectify representational deficiencies in various fields by positive discrimination, EDI initiatives promote changes in human resource practices, such as training in ‘unconscious bias’, and concrete measures and goals for improvement, some of which are hard won via legal challenges [35]. EDI affects sex and gender science by changing *who* is at the table or in the laboratory, and often what is proposed for study. Efforts to increase participation of women and racial minorities in higher education, science or medicine include the UK Athena Swan [36], Dimensions Canada [37], and the US Sea Change [38]. These initiatives are aimed at changing culture, gender and leadership in medicine and STEM(M) programs in general. EDI initiatives often rely on questions to determine group/identity membership, and in some cases, protect funding or opportunities for women, racialized, indigenous, sexual and gender minorities, and dis/abled individuals in science.

Both SGBA+ and EDI initiatives are policies that rely on solid evidence and can also lead to a broader health research agenda. SGBA+ highlights issues and evidence gaps and can spur new research. EDI broadens research agenda and ways of thinking and knowing, by increasing diversity among researchers. Both initiatives undermine homogenous and categorical thinking and contribute to the growth of sex and gender science.

## 5. The Goal: Achieving Equity

Ultimately, the goal of all of these research and policy initiatives and specialties is the adoption of gender-transformative approaches that usurp both gendered power structures and inequities at the same time. Gender-transformative approaches in health require clarification of dual aims: improving health as well as improving gender equity at the same time [39]. Such goals transcend sex- and gender-specific modifications that focus on personalizing or tailoring health care or promotion without addressing the root causes of gender inequity that may be contributing to such. Where a sex- or gender-specific approach results in different interventions or processes based on a sex or gender category per se, gender-transformative approaches aim to address the underlying mechanisms that drive inequity. This calls upon health researchers, healthcare practitioners, policy makers and program developers to consider short- and long-term impacts of sex- and gender-related factors and to develop creative approaches and measures for reaching both gender equity and improved health.

Doing gender-transformative health research, policy making, program design, or care is creatively demanding, and requires outcome measures in gender equity as well as health, but ultimately represents a higher bar for evidence and action for health equity. It is important that transformative approaches aim to focus on sex- and gender-related factors and processes rather than sex/gender categories to achieve these goals, because a factor-oriented approach can not only better account for the heterogeneity within normative sex/gender categories and the overlap between them, but also account for the health realities of people with a range of gender identities and roles.

## 6. The Relationships among These Scientific and Policy Initiatives

These areas of research and policy activity are clearly linked and, in some cases, overlap or are mutually supportive, but are usefully distinguished in order to avoid muddiness in thought and action, and more effective implementation. Given a consistent history of conflation of sex and gender concepts, it is no surprise that these areas of sex and gender science and policy are also often conflated. 

For example, proclaiming and achieving EDI goals cannot and does not replace the need for SGBA+ but rather underscores the importance of understanding and applying SGBA+. Increasing the participation of women and girls or racialized groups in health research requires clear plans, programs and initiatives that reflect deficiencies in the evidence and policy gaps. Such programming best relies upon and integrates SGBA+ analyses of how science has not only channeled girls away from STEM(M), but also avoided researching issues that seek to explain the impact of these diversions. For example, research and treatment has not mirrored the sex distribution of cardiovascular disease (CVD) in the population or the problems of gendered clinical approaches to diagnosis of CVD, [40] or the impact of race/ethnicity on CVD, and the social factors affecting the distribution of CVD among subgroups such as blacks, south Asians, or indigenous women and men [41]. Such inequities can be partially addressed and redressed via SGBA+ and EDI together. Ultimately, all health researchers, funding organizations and governments need to grapple with EDI goals to broaden research agenda and measure outcomes and impacts of EDI. For example, increasing sex and gender content in health research is linked to diversity in research teams, and both are linked to success in funding [42].

Sex and/or gender differences research approaches are often important for developing signals of importance and can generate new questions or signify differential impacts on males and females/men and women/gender diverse people. However, without understanding the sex/gender-related factors and processes that underlie differences, our capacity for action and intervention is limited. In the same vein, intersectional approaches expand analyses but do not preclude the need for understanding sex and gender science, skill building in SGBA+ or developing mixed methods and comprehensive measures to incorporate a range of sex- and gender-related factors along with race-related, income, age, ability and other multi-level factors [43,44]. Research specifically focused on sexual and gender minority populations demands even more depth of understanding and analysis of sex and gender concepts to fully understand and document real world experiences connected to identities and behaviours that can be fluid.

Finally, the business of identifying gender transformative approaches that stem from sex and gender science remains a pinnacle, for what is the point in advancing sex and gender science without advancing gender and health equity? In order to actively pursue gender-transformative solutions, a sophisticated SGBA+ and an intersectional lens are paramount.

## 7. Conceptualizing and Discussing Sex/Gender

In all of these areas of activity, the ways in which we discuss sex and gender underpin and influence the ways we think about sex and gender (and concomitantly, our thinking influences the way we write and speak). Paying attention to these discourses of sex/gender and how they shape our science, policy or programming can help to open up the landscape of possibilities for how we address sex and gender in health research and policy and alleviate the tendency to rely heavily on a comparative binary for taking up these issues.

One aspect of this is to consider carefully what we mean by the terms—what is ‘sex’, and what is ‘gender’? For many years, it was commonplace for health researchers to treat them as simply synonyms, with gender often chosen because it avoided the sexualized connotations of sex. At times, gender has been a synonym for women in health policy and international development. The terms, sex and gender have often been conflated, exchanged or misused in research, care and policy, and there have been efforts to clarify these in research and reporting [34,42]. In recent decades, most health researchers and others working in health have been encouraged to employ a conceptual distinction between sex and gender that sees sex as principally about biology, while gender as principally about sociology. This is a useful and important toehold, but a blunt distinction and oversimplification, and does not encourage a full, nuanced picture of how sex- and gender-related factors are multi-faceted and interact with one another (and with other intersectional factors and processes) dynamically across the lifespan, reflecting their temporal and cultural contexts. There is no hard boundary between the body and the world: the biology of the body can and does have social implications, and social experiences can and do have concrete effects on biology [45,46] with short- and long-term impacts on physical and mental health [47]. The precise operationalization of these terms should be carefully considered and elucidated as appropriate to the research, bearing in mind its purpose and context.

This distinction between sex and gender has also led many biomedical researchers, particularly those using cell cultures or animal models in experimental designs, to dismiss gender as irrelevant to their work. Although it may be challenging or difficult to address gender-related factors directly through experimentation, basic biomedical researchers should not overlook the relevance of elements of gender for the ways in which their research is designed, questions are formulated, and how their findings are analyzed, interpreted or applied in both subsequent research, and in real life. While researchers engaging in this kind of modelling need to be deeply self-critical and attentive to the dangers of anthropomorphism and naturalistic fallacies, animal hierarchies, dominance, aggression and nurturing processes can be successfully manipulated along with access to socialization or resources, in order to simulate gendered and other social practices [48].

Similarly, many social science researchers have often ignored or denied the intricacies of sex-related factors, material biological realities of reproduction, and real-life sex-gender interactions in formulating research about gender and health. This can lead to reliance on gender theory to explain bodily changes and impacts, clearly limiting the scope of understanding sex-related factors and processes in science and in life. This can lead to some gender and health research being narrowly conceived and partial in developing understandings of how gender, and its interpretations, subjectivities and performativity, links to the material sexed realities of bodies and developmental processes to impact health.

In part because of social justice forces and the promulgation of the various and overlapping fields of endeavour articulated above, the concept of gender is often equally misunderstood and partially applied. Notably, in recent years it has become commonplace for the concept of gender to be narrowly understood as gender identity—the sense of one’s self as a gendered person. This is in large part due to the increases in visibility and social recognition of trans people and those with diverse gender identities, which has been a force pushing for gender equity in health care and research, often in concert with pursuing EDI-related goals of inclusion. However, it is crucial to note that gender is much more than just gender identity; it also encompasses gender roles, gender relations, and institutionalized gender, all of which interact with one another, affect all gender identities, and are temporal and culturally specific (Table 1) [49]. The conflation of ‘gender identity’ with ‘gender’ does not serve health research, care, policy or programming well, and impairs the development of a nuanced and comprehensive sex and gender science. Similarly, conflations of sexual identity with the concept of sex are equally inaccurate; indeed, sex is much more than sexual behaviour, expression or orientation.

## 8. Clusters Not Categories?

Many discourses of the sex/gender variables in health lean very heavily on the invocation and use of a male–female sex binary and/or a man/woman gender binary. This can be problematic because such a starting point not only shapes scientific practice along dichotomous lines, but can also obscure learning about the mechanisms, components, processes and influences that are responsible for sex/gender-related disparities in health. It can also exclude those with differences of sexual development or diverse gender identities. A binary approach can also be misleading as it masks and restricts our understanding of the wide range of characteristics, overlaps, life course changes and the heterogeneity of sex- and gender-related factors that affect health. 

One particularly concerning outcome of this approach is that it runs a high risk of mistreatment of individuals who do not conform to the group mean. In other words, “comparisons of males and females may not be generalizable to all males and all females” [51]. For example, after a higher rate of next-day drowsiness was reported among women using the sleep aid zolpidem, the Food and Drug Administration in the USA recommended dosing based on gender category [52]. Although a male–female comparison of zolpidem clearance indicated a lower apparent clearance rate of the drug in women 8 hours after dosing (the relevant time for the outcome of next-day drowsiness), there was no difference in concentrations of active drug or driving impairment. After probing beyond the male–female difference, Greenblatt and colleagues conclude that not only was gender-specific dosing not warranted, the reduced recommended dosage for women “may in fact lead to underdosing and the consequent hazard of inadequately treated insomnia” in women (p. 189, [52]).

In another example, Goldman et al. reported that women who were frequent blood donors were at higher risk of ferritin depletion than men [53]. On the basis of this male-female comparison, the Canadian Blood Services changed their donor recall criteria based on gender category, with women donors being limited to four donations per year, rather than six per year for men [53]. Although this change was well-intentioned and aimed to protect the health of female donors, it does not address any of the likely mechanisms that tended to put women at risk in the first place: body size, menstrual status, and dietary intake of iron are three substantial factors contributing to the observed male-female difference. Viewed in that light, it seems hard to justify treating a 90 kg woman who eats meat regularly and uses contraception that causes cessation of periods as though she has the same risk as a 55 kg vegan woman with regular heavy periods simply because they are both women and that a 70 kg vegan man has a lower risk than her simply because of his gender.

Conceptualizing males and females as distinct categories, as is common, sets up a pattern of thinking and analysis that treats these as separate, dichotomous, non-overlapping groups—encouraging “sex differences” research as the ultimate contribution, as opposed to it being seen as a gateway to further questions about biological factors and processes affecting health. Socially, it sets up the popularized ‘men are from Mars, women are from Venus’ phenomenon, where diversity within the sex/gender groups and the overlaps between them are overlooked. It is common for people to fall into conceptual traps such as “estrogen = women” or “testosterone = male”. Even contemporary textbooks of physiology reproduce and reinforce this problematic way of thinking [54] with chapter headings like “estrogen and other female sex hormones”, or describing testosterone as “the male sex hormone.” In reality, estrogen and testosterone serve important functions in all bodies throughout the lifespan and are hormones that are certainly “sex-related” but certainly not categorically “sex-specific.”

Gender, when utilized as a concept in health research, can often lead to similar categorizing instead of clustering, and reinforces a commonly understood man/woman boy/girl categorization of gendered experience. However, when broken down into the range of gender-related factors, including norms, roles, relations, identities and institutionalized gender in Table 1, a more complex set of factors and experiences can be identified and measured. For example, as mentioned, it is currently common for people to conflate gender and gender identity, but these equations betray a partial and erroneous understanding of gender. A more holistic understanding beyond gender identity alone enables an appreciation and examination of the full range of gender-related factors that affect all genders.

When considering a range of gender identity categories (for example, women, men, non-binary, trans, fluid, Two Spirit, Hijra, among many others past, present, and as yet unnamed) it is evident that the psychosocial adoption of “identity” can take on many forms and is not necessarily fixed or stable. Ultimately gender identity refers to the integration of, commitment to and expression of dominant gender ideologies and expectations which differ widely within and between all of these categories and their interaction with the material biological manifestations that go along with being a sexually reproducing species. Understood this way, gender identity is something everyone has developed in different forms, incorporating their gender expression in accordance with, or in resistance to, dominant gender ideologies that are temporal and (sub)cultural.

These messages are dominantly transmitted via socialization processes that operate from micro to macro levels and serve to reinforce each other in (sub)cultural contexts. These processes may result in specific behaviors and attitudes such as: adoption of forms of gendered dress and performance (mode, colours, styles); presentations of self (body posture, attitude, space); ways of speaking (tone, authority, volume, content), body movement (sitting; standing, running, walking); and ones’ psychosocial congruence with and level of adherence to culturally understood and approved dominant masculine and feminine qualities. For example, transmasculine or transfeminine people, transmen or transwomen, or masculine women or feminine men or feminine women or masculine men are all claiming identities or having identities thrust upon them, or both, and are interacting dynamically with their social environments via their assumptions of elements of femininities and/or masculinities.

Gender roles and norms are also key factors for health researchers to consider. For example, among other traits, caretaking and caregiving are dominantly understood to be feminine/women’s roles and are encouraged and socialized accordingly. Masculinities are dominantly understood to include self-reliance, risk taking and limited emotional expression, among other traits. Interrogating these roles in the context of health is crucial. Do they contribute to men’s reduced help seeking, higher uses of substances, rates of dying alone from opioid overdose or suicide? Do they impede women’s healthy behaviours and health care seeking for themselves when women often put others’ health and nutrition and relaxation before their own? Do they impact self-assessment to the point of denying themselves such treatments as a joint replacement, and/or affect clinicians to the point of not offering one? [55,56].

Gender relations are similarly impactful on health. The power distributions, and decision-making scope in romantic, sexual, household or work relationships are often gendered, and affect how individuals can pursue health or access health care. In what ways do couple dynamics contribute to initiation and maintenance of substance use, for example? How does a lack of autonomy, freedom of movement or culturally condoned intimate partner violence affect people of different genders (for example, the increased risk of IPV for women and people with diverse gender identities, the ways that normative ideologies of masculinity fuel many dynamics in IPV, and the gendered forms of stigma experienced by men who are victims of IPV)? How do heteronormative assumptions in health care provision deny the relational component of illness or healing for gender diverse people or non-heterosexual patients?

## 9. Clarifying the Questions

Integrating a more conscious recognition of sex- and gender-related factors in health research and ultimately in health care and policy is critically important in providing personalized care and achieving equity. Subtle changes in the language we use to discuss sex/gender and health can have potentially profound impacts on both research practice, health care practice and consumer literacy. For example, in asking the question, “what is the influence of sex and gender on immune function?” it is very common to frame the discussion in terms of male vs. female comparisons and launch a search for sex/gender differences. However, if we frame the question as “what are the influences of sex- and gender-related factors on immune function?”, it directs our attention to the interactive factors that may mechanistically explain male–female or men/women/gender diverse group differences, and thereby avoid fostering the idea that ‘sex’ and/or ‘gender’ are themselves a ‘cause.’ Furthermore, it allows a more robust assessment of both the biology and sociology associated with patterns, trends and impacts.

A key example of the importance of studying using a frame of ‘sex-related factors’ is illustrated by the pharmacokinetics of drugs, which are determined and affected by a range of sex-linked, or -specific or -affected processes such as absorption, distribution, metabolism and elimination of drugs [57]. These processes can be affected by things like hormone levels, enzyme expression, body composition, body size (weight and height), organ size, and age. The net result of this complex set of processes and characteristics can be that sex- and gender-related factors may be relevant considerations in determining appropriate dosages. However, that does not necessarily mean that dosing should be based solely on a sex or gender category. The impact of a wide set of sex- and gender-related factors on real world experiences after ingesting prescribed or recreational drugs is an everyday example of the importance of going beyond sex category in understanding physiology and informing regulations, interventions and policy. Asking further questions about how ‘sex- and gender-related factors interact’ to impact people after drug ingestion would quickly reveal a wide set of health impacts that include: intoxication or overdosage; gendered stereotypes that affect the prescription and/or use of drugs; impacts of gender norms on misuse, overuse and risk-taking; gendered relationship dynamics that affect initiation, reduction and recovery; and sexual assault, STIs and other harms that can result that are sex/gendered in impact. 

Further, reframing our questions to include ‘factors’ and sex and gender is not only more appropriate, but also more scientifically useful, informative, and actionable, and helps move us to a more gender transformative mode of research, discovery, innovation, and policy-making. Such a model incorporates goals of moving toward health equity and gender equity at the same time, by assessing how sex-/gender-related factors are overlooked (gender-blind and gender-neutral), accommodated (gender-specific and gender-sensitive) or actively changed to drive equity (gender-transformative). While social action may seem distal to an animal science experimenter, these activities are intrinsically linked, and therefore discourses surrounding lab-based experimentation matter. Similarly, while biological mechanisms and processes may seem distal to a social science researcher, they are critically important to understanding the health and wellbeing of every body. Ignoring one aspect in either case, is to ignore reality, real world experiences and inequities. Cross- and trans-disciplinarity is vital to progressing sex and gender science [13].

## 10. Knowledge Translation

The various fields of endeavour outlined above: sex and gender science, its subsets sex/gender differences, sex and gender related factor research, sex and gender interactions research, sexual and gender minority research, along with intersectional analyses, SGBA+ processes, EDI initiatives and gender transformative approaches are clearly related in their purposes to uncover more knowledge about sex-, gender- and equity-related factors and health, to create conditions for more gender equity, and inasmuch as they are overlapping can be seen as having common cause. Their overarching purpose is to achieve more precision and understanding of relevant interactions and intersectional factors to achieve better health, health care and increased gender equity. When considering these overarching goals, it is important to reflect on language, purpose, audience and cultural context. 

When interpreting and reporting on sex-related factors or gender-related factors, it is important to reflect on heterogeneity as well as homogeneity, changes over lifespans and developmental phases, and overlapping processes, along with primarily sex- and gender-specific patterns and characteristics. For example, when experimental and analytical design is framed primarily on a male-vs-female comparison (sex differences), we have a priori predicted and supported a continuation of dichotomies and categories and limit our ability to develop alternative interpretations and considerations. This can lead to overestimating the importance of differences in means, and underappreciating the overlap between groups, as illustrated by the zolpidem and blood donation examples above. 

It is also important to consider precision not only in our approaches to health care and research and equity, but also in language. Gender-neutral and gender-blind language may seem superficially “inclusive”, but it is completely unhelpful in supporting precision care, creating and maintaining accurate data sets, advancing science, fine tuning our collective understanding of sex- and gender-related factors and health, or for making or keeping sex/gender inequities visible. Similarly, educating each other as researchers, practitioners and policy makers about the details of various sex- and gender-related factors, and real-world experiences of health and health care is crucial to better knowledge translation, mobilization and implementation. To fully explore sex–gender interactions and intersectional impacts requires continued efforts to develop complex methods and measures regarding sex and gender, and more, not less precise language and terminology in research, policy, practice and administrative contexts. 

This also requires more consideration of multi and mixed methods in health research, and increased appreciation and understanding of transdisciplinary approaches and methods. Adopting a transdisciplinary approach to health research is the ultimate ideal for engaging more effectively with sex- and gender-related factors, and in so doing, developing a solid bridge between biology and sociology. 

Most importantly, our audiences. To improve consumer health literacy and understanding of health, whether among women, men or gender-diverse people, will require more precise language and conceptualizations and a deeper understanding of the ever-changing constellations of sex-, gender- and equity-related factors that are affecting our own bodies and experiences. Without these, groups requiring and seeking gender equity and health equity will remain unarmed, acritical, under informed, unhealthy, and possibly unsafe, and gender-blind equity frameworks will continue to proliferate [58]. Similarly, clinicians of all kinds will benefit from assistance in providing more precise health care if they are armed with more information, knowledge and guidance that is sex and gender sensitive and equity oriented. Without a continued commitment to linguistic and conceptual precision and data collection, both sex and gender science and health care will suffer, thereby setting back, not advancing, our mutual enterprises.

## 11. Conclusions

Current proliferations of several related fields of endeavour in sex and gender science can benefit from clarification of scope and purpose. Without a shared understanding of the various enterprises aiming at increasing gender and health equity, there is a risk of subsuming or obliterating past gains by newer political and scientific initiatives and goals. As sex and gender science evolves, more precision is required in discussing and applying concepts; mapping the relationships, purposes and overlaps between research and policy initiatives; and building our collective knowledge of sex- and gender-related factors affecting health. Doing so will deepen our understanding of the important ways in which sex and gender affect health, health care and medicine. Without sharing and committing to these common objectives, efforts to progress sex and gender science risk being diminished, delayed, conflated and confusing, and gender-transformative solutions to health inequities will recede further into the distance.

## Figures and Tables

**Table 1:** Gender identity, roles, relations, and institutions.

Gender Identity	How individuals see and situate themselves within the landscape of masculinities, femininities and gender identities available in their cultural and temporal context. Gender identity develops in a social milieu where there are typically strong, clear messages about gender norms and presentations, and a person’s outward expressions of their gender identity can be influenced by the extent to which that identity is affirmed or discouraged in that milieu.
Gender Roles	The culturally expected and approved norms of behaviour that are expected from individuals based on their sex class and gender identity, and which influence an individual’s actions, opportunities, domestic and family roles and responsibilities, access to resources, and occupational and educational roles.
Gender Relations	The ways in which individuals interact with one another, differentially experience authority, autonomy, freedom of movement and decision-making power and are treated by others based on an ascribed gender category in relationships of various kinds (including romantic, sexual, domestic, family, household, work or other relationships).
Institutionalized Gender	The many ways in which power and resources are differentially distributed in political, cultural, educational, religious, media and social institutions in a society to define, reproduce, and justify the differential treatment and power afforded to people based on sex and/or gender. Institutional gendered factors often remain unseen and their impacts invisible, such as channeling people into certain professions, stigmatizing certain behaviours, promoting gendered imagery, providing unequal pay based on sex, gender identities and roles and inducing individual and group compliance with normative cultural expectations about gender.

Updated based on Johnson, Greaves and Repta, Better Science with Sex and Gender: A Primer for Health Research, 2007 [50].
